# A retrospective study on the prevalence and genetic characteristics of porcine parvovirus 6 in Guangxi, China

**DOI:** 10.3389/fmicb.2026.1754811

**Published:** 2026-01-27

**Authors:** Liang Cao, Chenxi Ji, Ziping Yu, Wei Wang, Lulu Kang, Lin Jin, Zaiyong Han, Fulong Nan, Wenjie Li, Jialiang Xin

**Affiliations:** 1 School of Laboratory Medicine, Jilin Medical University, Jilin, China; 2 Institute of Virology, Wenzhou University, Wenzhou, China; 3 College of Veterinary Medicine, Qingdao Agricultural University, Qingdao, China; 4 Key Laboratory of Animal Disease and Human Health of Sichuan Province, College of Veterinary Medicine, Sichuan Agricultural University, Chengdu, China

**Keywords:** amino acid mutation, co-infection, genetic evolution, porcine circovirus, porcine parvovirus 6, selection pressure

## Abstract

Porcine parvovirus 6 (PPV6) is an emerging virus whose epidemiology and clinical significance in China remain poorly defined. This study investigated the prevalence and genetic characteristics of PPV6 in Guangxi Province, China, using 497 porcine serum samples collected between 2015 and 2019. The overall PPV6 prevalence was 12.5% (62/497), with regional rates ranging from 12.0 to 17.6%. A high frequency of co-infection with porcine circoviruses (PCVs) was detected, with 46.8, 19.4, and 3.2% of PPV6-positive samples were also positive for PCV2, PCV3, and PCV4, respectively. To the best of our knowledge, this study describes the first reported detection of PPV6 and PCV4 co-infection. Ten complete PPV6 genomes were successfully sequenced and grouped into two sizes, 6,112 and 6,111 nt, the latter resulting from a single thymine deletion in the 3’ untranslated region (UTR). Phylogenetic analysis based on VP1 sequences classified global PPV6 strains into two distinct lineages (A and B). All strains identified in this study clustered within Group B. Conversely, three previously reported Chinese strains belonged to Group A. Fourteen amino acid substitutions in VP1 were strongly associated with this phylogenetic separation. Selection pressure analysis further identified multiple positively selected sites within the capsid protein. Overall, these findings advance understanding of the molecular epidemiology of PPV6 in southern China and highlight its potential interaction with PCVs, providing a basis for future investigations into viral pathogenesis and vaccine development.

## 1 Introduction

Parvoviruses are small, non-enveloped viruses possessing a single-stranded DNA genome of approximately 4–6.3 kb, encoding two primary open reading frames (ORFs) (Oh et al., 2017). ORF1 encodes the non-structural protein NS1, which is essential for viral replication, while ORF2 encodes the viral capsid protein (VP1) (Oh et al., 2017). The genus *Copiparvovirus*, which includes several porcine parvoviruses (PPVs), has received increasing attention due to its potential association with clinical disease in pigs (Streck and Truyen, 2020; van Leengoed et al., 1983).

Since its initial identification in aborted pig fetuses in China, porcine parvovirus 6 (PPV6) has been detected in swine populations in several countries, including the United States, Poland, and Mexico (Cui et al., 2017; Garcia-Camacho et al., 2020; Ni et al., 2014; Schirtzinger et al., 2015). Reported prevalence varies by pig demographic, with higher detection rates in aborted fetuses and piglets compared with sows and finishing pigs (Ni et al., 2014). A significant aspect of PPV6 epidemiology is its frequent co-infection with other porcine viruses, particularly porcine circoviruses (PCVs) (Andersson et al., 2011). Studies suggest that PPVs may function as co-factors in porcine circovirus-associated disease (PCVAD) by enhancing porcine circovirus 2 (PCV2) replication. For instance, PPV6 has been detected at significantly higher rates in PCV2-affected farms than in unaffected ones (Milek et al., 2020). Similar interactions have been observed with porcine circovirus 3 (PCV3), with studies reporting high co-infection rates involving PPV6 and porcine parvovirus 7 (PPV7) (Ha et al., 2018).

PPV6 was first reported in China in 2014, and was subsequently identified in multiple regions, including Heilongjiang Province in Northeast China, Beijing Municipality and Shandong Province in North China, Sichuan Province in Southwest China, and Guangdong Province in South China (Ni et al., 2014; Zhao et al., 2024). However, epidemiological data on PPV6 prevalence remain limited for Guangxi Province, also located in South China. PCV4 was first identified in Hunan Province in 2020, and its prevalence and co-infection patterns have yet to be fully characterized (Zhang et al., 2020). This study aimed to address these gaps by determining the prevalence of PPV6 in Guangxi Province, assessing co-infection with PCV2, PCV3, and PCV4, and performing a comprehensive genetic and evolutionary analysis of circulating strains.

## 2 Materials and methods

### 2.1 Sample collection

**FIGURE 1:**
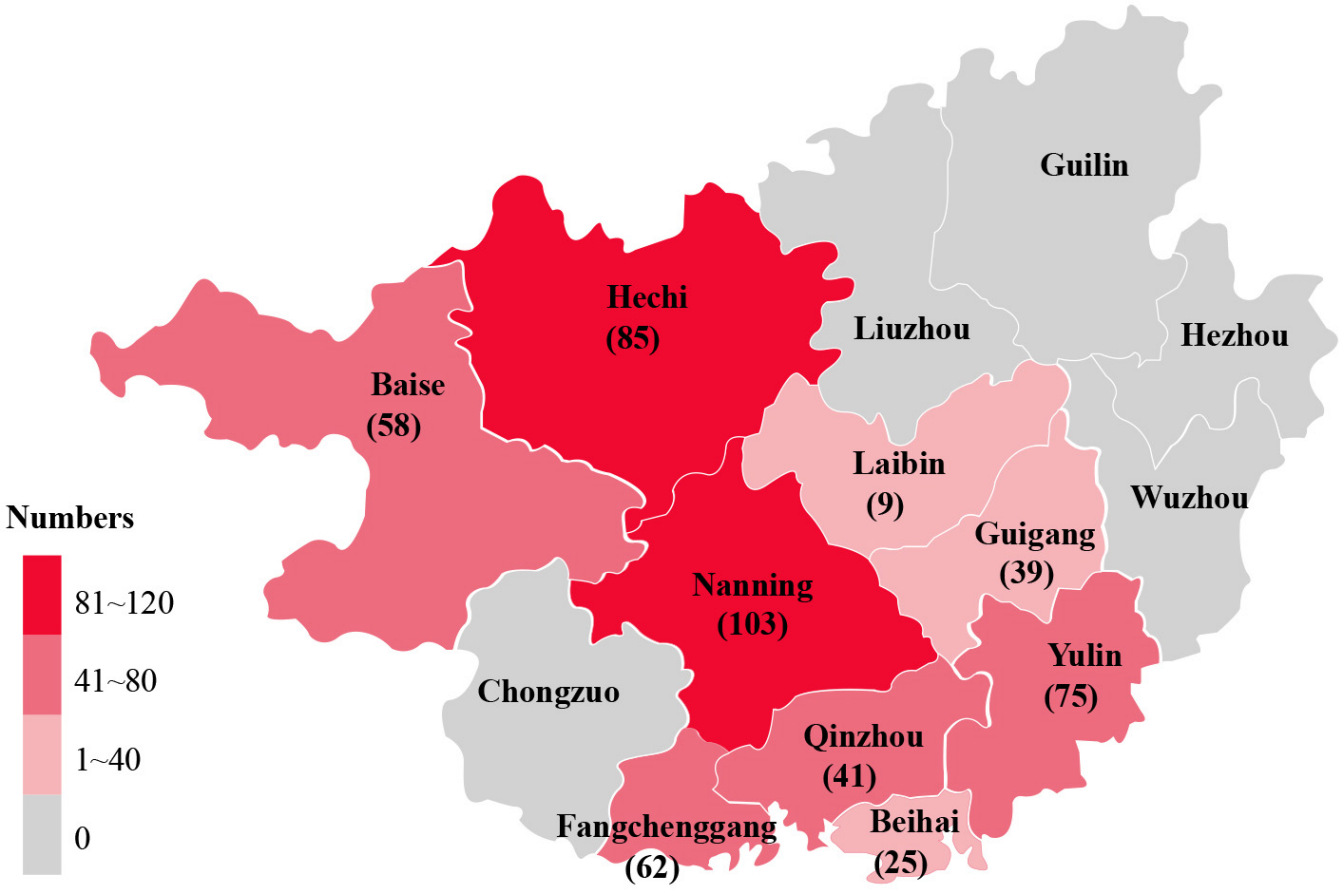
Geographical distribution of serum samples collected from Guangxi, China.

A total of 497 serum samples were collected from pigs aged 2–17 weeks across nine cities in Guangxi Province (Nanning, Baise, Yulin, Fangchenggang, Beihai, Qinzhou, Guigang, Hechi, and Laibin) between 2015 and 2019 (Figure 1). Samples were provided by the Guangxi Center for Animal Disease Control and Prevention and stored at -80°C until analysis.

### 2.2 DNA extraction and polymerase chain reaction detection

Viral DNA was extracted from 100 μL of serum using the TIANamp Virus DNA Kit (TIANGEN, Beijing, China), according to the manufacturer’s instructions. Specific primer pairs were designed based on available PPV6 sequences deposited in GenBank (Supplementary Table 1). PCR amplification was performed using 2 × Phanta Flash Master Mix (Vazyme, Nanjing, China). The thermal cycling conditions consisted of an initial denaturation for 3 min at 95°C, followed by 35 cycles of denaturation for 15 s at 95°C, annealing for 15 s at 60°C, extension for 30 s at 72°C, and a final extension for 5 min at 72°C. The amplification products were cloned, and positive amplicons were sequenced by Sangon Biotech (Shanghai, China).

### 2.3 Genome assembly and sequence analysis

Complete genomes of ten PPV6 isolates were assembled from the sequenced fragments and deposited in GenBank under accession numbers OM811448–OM811457. Sequence alignment and similarity analyses were performed using Molecular Evolutionary Genetics Analysis (MEGA) 7.0 (Kumar et al., 2016). The RNA secondary structures of the 5’ and 3’ untranslated regions (UTRs) were predicted using the RNAFold Web Server^1^ (Mattei et al., 2015).

### 2.4 Selection pressure analysis

Selection pressure acting on VP1 was assessed using the Datamonkey Web Server for Molecular Evolution Analysis^2^ (Weaver et al., 2018). Four analytical methods were applied: Single-Likelihood Ancestor Counting (SLAC), Fixed Effects Likelihood (FEL), Fast Unconstrained Bayesian Approximation (FUBAR), and Mixed Effects Model of Evolution (MEME). Sites were considered to be under positive selection if they met one of the following criteria: *p* < 0.1 for SLAC, FEL, or MEME, or posterior probability > 0.9 for Fast Unconstrained Bayesian Approximation (FUBAR).

### 2.5 Phylogenetic analysis

Phylogenetic trees based on NS1 and VP1 amino acid sequences were reconstructed using MEGA version 7.0. The Neighbor-Joining (NJ) method was used to infer relationships within the subfamily *Parvovirinae*; the Maximum-Likelihood (ML) method, employing the JTT + G + I model, was used to reconsturct PPV6 phylogeny. Node robustness was evaluated using 1,000 bootstrap replicates (Zhao et al., 2024). Reference sequences of PPV6 and related parvoviruses were retrieved from GenBank (Supplementary Table 2).

### 2.6 Statistical analysis

All data were analyzed using two-tailed statistical tests to assess significance. Statistical significance was set at *P* < 0.05. All statistical analyses were performed using SPSS Version 26.0 (SPSS Inc., Chicago, IL, United States).

## 3 Results

### 3.1 Prevalence and co-infection of PPV6

Among the 497 serum samples analyzed, 62 were PPV6-positive (12.5%), and 435 were PPV6-negative (87.5%). Geographic variation in prevalence was observed, with the highest rate in Hechi (17.6%, 15/85) and the lowest in Beihai (12.0%, 3/25). Comparative analysis of PCV infection between PPV6-positive and PPV6-negative groups showed significant differences:

- The PCV2 positivity rate was significantly higher in the PPV6-positive samples (46.8%, 29/62) than in PPV6-negative samples (14.9%, 65/435) (χ^2^ = 35.85, df = 1, *P* < 0.001).
- Similarly, PCV3 positivity was substantially greater in the PPV6-positive group (19.4%, 12/62) compared with the PPV6-negative group (4.6%, 20/435) (χ^2^ = 19.62, df = 1, *P* < 0.001).
- Although the prevalence of PCV4 was higher among PPV6-positive samples (3.2%, 2/62) than among PPV6-negative samples (1.6%, 7/435), this difference was in significant (χ^2^ = 0.798, df = 1, *P* > 0.05).

Overall, 69.4% (43/62) of PPV6-positive samples were co-infected with at least one PCV, a proportion significantly higher than that observed in PPV6-negative samples (21.1%, 92/435) (χ^2^ = 63.74, df = 1, *P* < 0.001) (Table 1).

**TABLE 1:** Detection of PPV6 co-infections with PCV2, PCV3, and PCV4 in serum samples isolated from eight cities in Guangxi, China.

Sample source	Numbers	Simplex infection				Co-infection		
		PPV6	PCV2	PCV3	PCV4	PPV6+PCV2	PPV6+PCV3	PPV6+PCV4
Nanning	103	13	15	5	2	7	3	1
Baise	58	6	4	2	1	2	1	0
Yulin	75	9	9	1	0	6	3	0
Guigang	39	7	3	0	0	3	0	0
Fangchenggang	62	5	8	3	1	0	1	0
Beihai	25	3	4	2	0	1	1	0
Qinzhou	41	4	6	2	1	2	0	1
Hechi	85	15	13	5	2	8	3	0
Laibin	9	0	2	0	0	7	3	1
Total	497	62	65	20	7	65	20	7

### 3.2 Genomic characteristics of PPV6 isolates

**FIGURE 2:**
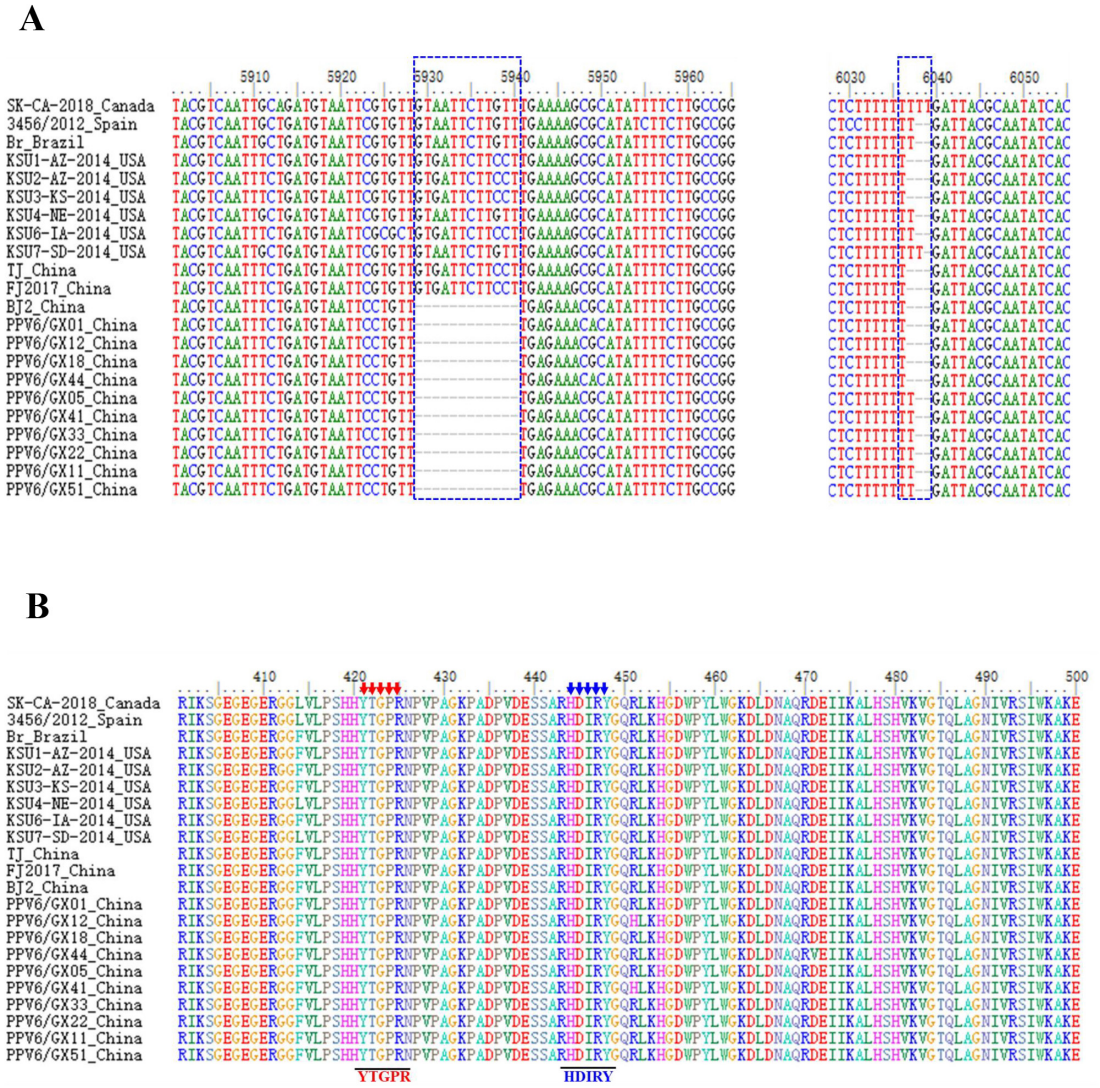
Sequence alignment of PPV6 isolates. **(A)** Alignment of nucleotide sequences representing different PPV6 isolates. **(B)** Putative phospholipase A2 (PLA2) motifs of PPV6 with reference parvovirus strains, including the Ca^2+^-binding loop (YTGPR) and catalytic residues (HDIRY).

Ten complete PPV6 genomes were successfully obtained. Six isolates were 6,112 nucleotides (nt) in length, whereas four were 6,111 nt owing to a single thymine deletion in the 3’ UTR. All isolates exhibited a conserved genomic organization, with ORF1 encoding a 663-amino-acid NS1 protein, and ORF2 encoding a 1,190-amino-acid VP1 protein. Sequence variation was primarily detected in the 3’ UTR, where four isolates had a 15-nt deletion, and six contained a 14-nt deletion relative to the reference strain, resulting in altered predicted RNA secondary structures (Figure 2A; Supplementary Figure 1).

Across the ten isolates, complete genome nucleotide similarity ranged from 98.9 to 99.6%. The ORF1 gene was more conserved (99.4–100% similarity among isolates; 98.8–99.8% relative to reference strains) than ORF2 (98.4–99.2% among isolates; 95.1–99.5% relative to reference strains). Critical functional motifs within VP1, including the Ca^2+^-binding loop (YTGPR) and the catalytic center (HDIRY) of phospholipase A2 (PLA2), were conserved entirely across all isolates (Figure 2B).

### 3.3 Positive selection analysis

Analysis of the VP1 identified multiple sites under positive selection. The MEME, FEL, FUBAR, and SLAC methods identified 16, 10, 18, and 2 positively selected sites, respectively. Seven sites (19, 113, 116, 163, 868, 869, 1,038) were supported by at least three methods. Notably, residue 103 was consistently identified as being under positive selection by all four methods (Table 2).

**TABLE 2:** Identification of positively selected sites (amino acid residues) in VP1 using codon substitution models.

Methods	Site
MEME	19, 38, 103, 116, 131, 133, 163, 241, 318, 360, 645, 791, 792, 868, 869, 1,038
FEL	19, 26, 36, 103, 116, 133, 163, 868, 869, 1,038
FUBA	5, 19, 23, 26, 36, 103, 116, 118, 127, 133, 163, 318, 324, 330, 645, 868, 869, 1,038
SLAC	103, 168

### 3.4 Phylogenetic and substitution analysis

**FIGURE 3:**
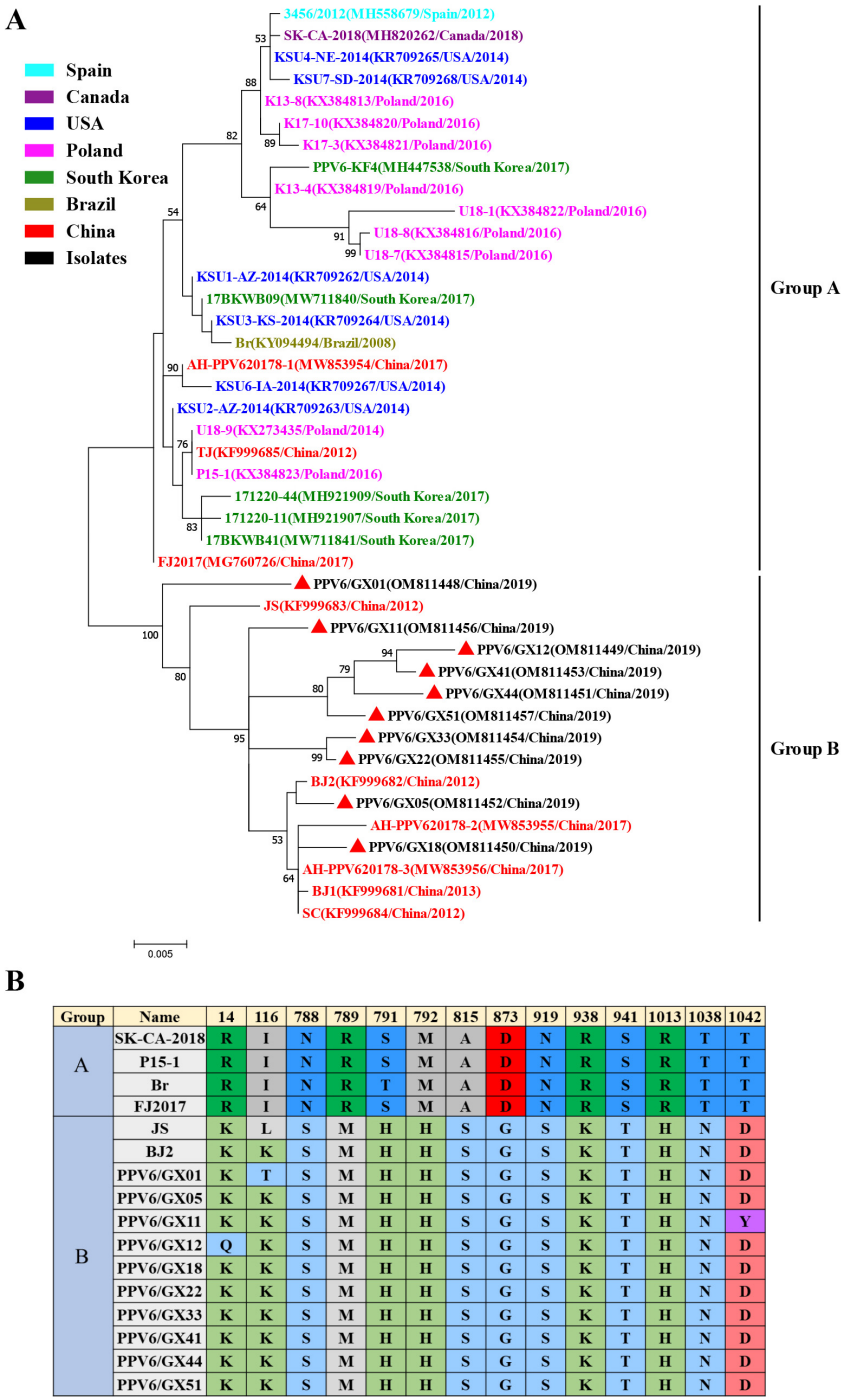
Phylogenetic analysis of PPV6 isolates. **(A)** Maximum-likelihood phylogenetic tree based on VP1 amino acid sequences of PPV6. Red triangles (π) indicate strains identified in this study. Bootstrap values (1,000 replicates) < 50% are not shown. **(B)** VP1 amino acid substitutions associated with phylogenetic clustering, showing differentiation between Groups A and B.

Phylogenetic analysis based on VP1 amino acid sequences separated global PPV6 strains into two distinct clades, designated Groups A and B (Figure 3A). All Guangxi isolates obtained in this study, together with most previously reported Chinese strains, clustered within Group B. Conversely, three earlier Chinese strains (AH-PPV620178-1, TJ, FJ2017) clustered within Group A, which primarily comprises strains from other geographical regions.

A total of 14 amino acid substitutions in VP1 that were strongly associated with this group differentiation: R14K/Q, I116L/K/T, N788S, R789M, S/T791H, M792H, A815S, D873G, N919S, R938K, S941T, R1013H, T1038N, and T1042D/Y (Figure 3B). Phylogenetic analysis based on NS1 amino acid sequences further confirmed that all isolates belong to the genus *Copiparvovirus*, and are closely related to PPV4 and PPV5 (Supplementary Figure 2).

### 3.5 Amino acid substitution patterns in VP1 and NS1

**FIGURE 4:**
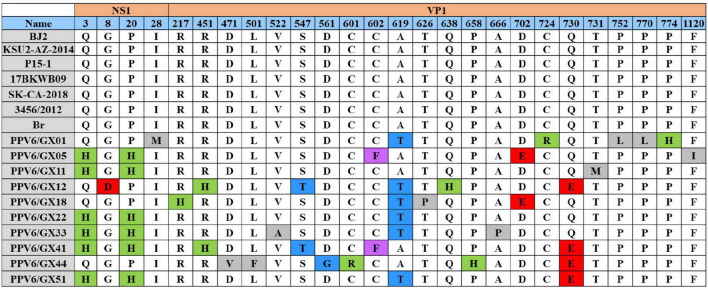
Amino acid substitution analysis of PPV6 isolates. Substitutions within putative conserved regions of NS1 and VP1 are shown.

Consistent with sequence similarity analysis, VP1 exhibited greater amino acid variability than NS1. Two substitutions (Q3H and P20H) were identified in the NS1 protein of most Guangxi isolates. Comparison of VP1 sequences with a reference strain revealed three major conserved regions (amino acid positions 164–256, 414–787, and 1,090–1,190), interspersed with more variable regions. Substitutions were distributed across all three conserved regions of VP1. Two substitutions, A619T and Q730E, located within conserved regions, were detected in the majority of Guangxi isolates, and may represent adaptive changes affecting functional specificity or pathogenic potential of local PPV6 strains (Figure 4).

## 4 Discussion

This study provides a comprehensive overview of the molecular epidemiology of PPV6 in Guangxi, China. The observed detection rate of 12.5% confirms active circulation of PPV6 within the local swine population. A PPV6 prevalence of approximately 13% has been reported in slaughtered pigs (Zhao et al., 2024), and the close agreement with our results indicates that PPV6 prevalence in pig populations has remained relatively stable, without evidence of short-term outbreaks or disappearance. Significantly higher positivity rates of PCV2, PCV3, and PCV4 were observed in PPV6-positive samples compared with PPV6-negative samples (*P* < 0.05), indicating a potential clinical association. These results are consistent with earlier reports demonstrating that PPV co-infections may enhance the severity of PCVAD (Allan et al., 1999; Kim et al., 2022; Milek et al., 2020). To the best of our knowledge, the identification of PPV6-PCV4 co-infection represents the first such report and warrants further investigation into its biological and clinical significance.

The genomic variations identified in this study, particularly deletions within the 3’ UTR that alter predicted RNA secondary structures, are of particular interest. Comparable UTR variations in other parvoviruses have been associated with regulatory functions influencing viral gene expression (Li, 1993). Whether the observed 3’ UTR deletions affect PPV6 replication or pathogenicity remains to be determined and will require functional validation using a reverse genetics system to characterize the role of the deleted region. The high conservation of the PLA2 motifs (YTGPR and HDIRY) was fully conserved across all isolates, underscoring their essential role in viral infectivity, as demonstrated for other parvoviruses (Dorsch et al., 2002; Zadori et al., 2001).

Phylogenetic analysis revealed that PPV6 strains segregate into two major groups (Groups A and B), driven by specific VP1 amino acid substitutions, suggesting ongoing viral evolution. The predominance of Chinese strains within Group B may reflect geographical clustering; however, the presence of three Chinese strains within Group A indicates either multiple historical introductions or convergent evolutionary processes. The greater variability observed in VP1 compared with NS1 is consistent with VP1 being the primary target of host immune pressure (Streck et al., 2015).

Finally, the identification of positively selected sites within VP1, including the consistently supported residue at position 103, highlights regions potentially involved in viral adaptation to host immune responses or other selective forces. Recurrent substitutions (A619T and Q730E) in VP1, together with NS1 substitutions (Q3H and P20H), may represent signatures of local viral adaptation and should be monitored in future molecular surveillance studies.

## 5 Conclusion

In summary, this study confirms the presence and genetic diversity of PPV6 in Guangxi, China. A relatively high prevalence of PPV6 was observed, together with frequent co-infection with PCV2, PCV3, and PCV4. Genomic analyses revealed significant variation in the 3’ UTR and VP1, with evidence of positive selection acting on the latter. The phylogenetic distinctiveness of local strains and the identified amino acid substitutions provide a molecular basis for future investigations into PPV6 pathogenesis, as well as the development of targeted diagnostic tools and vaccines.

## Data Availability

The datasets presented in this study can be found in online repositories. The names of the repository/repositories and accession number(s) can be found in the article/Supplementary material.

## Footnotes

1
http://rna.tbi.univie.ac.at/

2
http://www.datamonkey.org/

## Supplementary material

SUPPLEMENTARY FIGURE 1 Predicted RNA secondary structures based on untranslated regions of the reference and representative PPV6 isolates. **(A,B)** RNA secondary structures of the 5’ **(A)** and 3’ UTRs (B) of the BJ2 strain. **(C,D)** RNA secondary structures of the 5’ **(A)** and 3’ UTRs **(B)** of the TJ strain. **(E,F)** RNA secondary structures of the 5’ **(C)** and 3’ UTRs **(D)** of the PPV6/GX01 strain. **(G,H)** RNA secondary structures of the 5’ **(E)** and 3’ UTRs **(F)** of the PPV6/GX11 strain.

SUPPLEMENTARY FIGURE 2 Phylogenetic relationships of PPV6 within the subfamily *Parvovirinae.* The phylogenetic tree was inferred from NS1 amino acid sequences using the Neighbor-Joining (NJ) method with 1,000 bootstrap replicates. Red triangles (π) indicate strains identified in this study.
