# Supplementary figures and images for: A retrospective study on the prevalence and genetic characteristics of porcine parvovirus 6 in Guangxi, China
Source: Front Microbiol. 2026 Jan 27;17:1754811. doi: 10.3389/fmicb.2026.1754811 (PMC12886342; doi:10.3389/fmicb.2026.1754811)

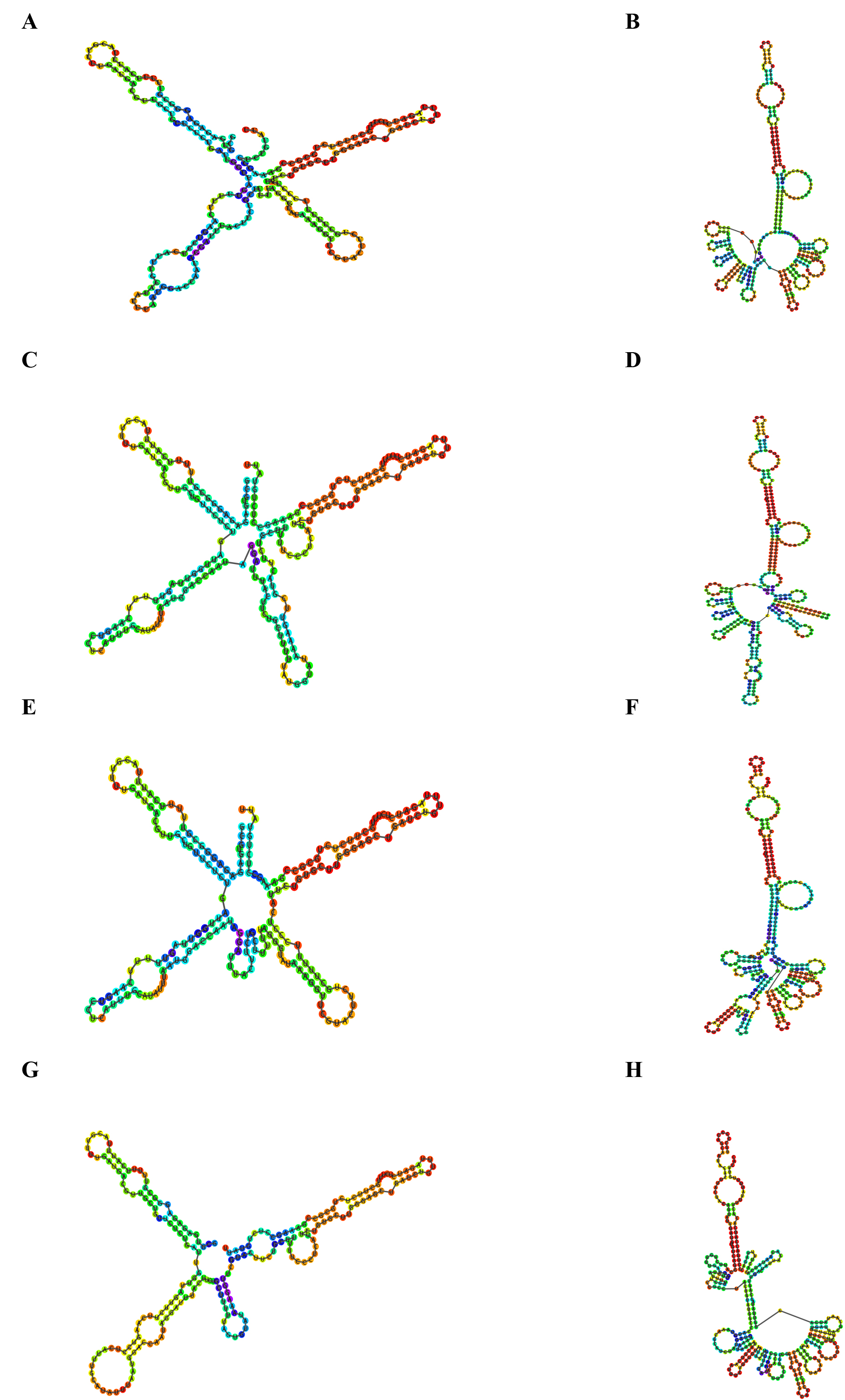

Supplement: SUPPLEMENTARY FIGURE 1 — Predicted RNA secondary structures based on untranslated regions of the reference and representative PPV6 isolates. (A,B) RNA secondary structures of the 5’ (A) and 3’ UTRs (B) of the BJ2 strain. (C,D) RNA secondary structures of the 5’ (A) and 3’ UTRs (B) of the TJ strain. (E,F) RNA secondary structures of the 5’ (C) and 3’ UTRs (D) of the PPV6/GX01 strain. (G,H) RNA secondary structures of the 5’ (E) and 3’ UTRs (F) of the PPV6/GX11 strain. [file Image_1.jpeg]

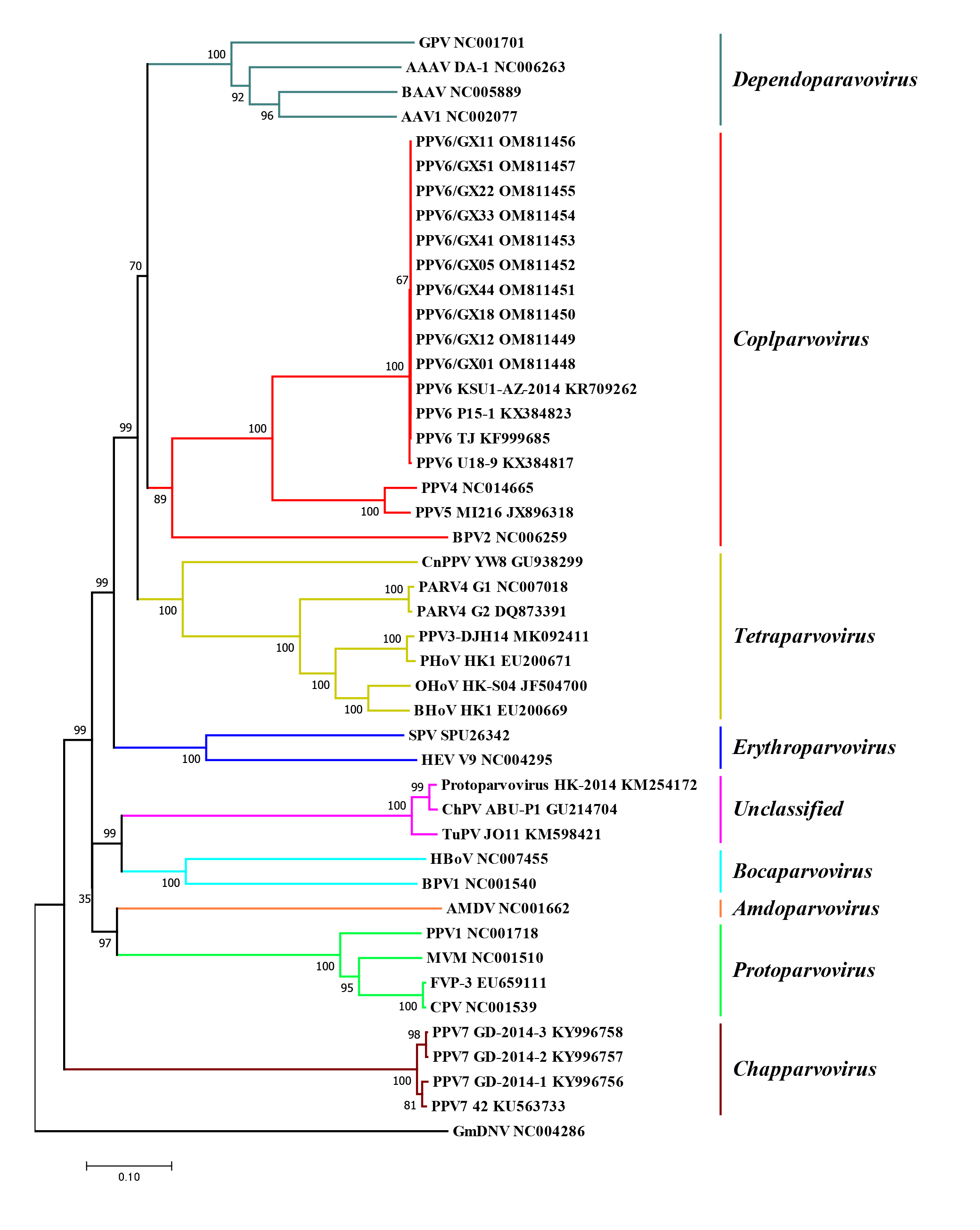

Supplement: SUPPLEMENTARY FIGURE 2 — Phylogenetic relationships of PPV6 within the subfamily Parvovirinae. The phylogenetic tree was inferred from NS1 amino acid sequences using the Neighbor-Joining (NJ) method with 1,000 bootstrap replicates. Red triangles (π) indicate strains identified in this study. [file Image_2.jpeg]
