# Supplementary material for: A retrospective study on the prevalence and genetic characteristics of porcine parvovirus 6 in Guangxi, China
Source: Front Microbiol. 2026 Jan 27;17:1754811. doi: 10.3389/fmicb.2026.1754811 (PMC12886342; doi:10.3389/fmicb.2026.1754811)
Supplement: Supplementary file 3 [file Table_1.docx]

**Table S1**. Primer sequences used in this study

| **Primer** | **Sequence 5’-3’** | **Position** | **（****Amplicon Length）bp** |
| --- | --- | --- | --- |
| PPV6-1F | GCGTGAGAGAGGGCGTTTTTCATTT | 1～337 | 337 |
| PPV6-1R | TTTTGCCCATTACGGGTACTCAC |  |  |
| PPV6-2F | GTGAGTACCCGTAATGGGCAAAA | 315～1783 | 1469 |
| PPV6-2R | CGAGAAGCAGAGGGAGAAGGAGA |  |  |
| PPV6-3F | TCTCCTTCTCCCTCTGCTTCTCGCCGC | 1761～3094 | 1334 |
| PPV6-3R | GACTTGACATCTGCCCTTCTTGTTTAGA |  |  |
| PPV6-4F | AAACAAGAAGGGCAGATGTCAAG | 3070～4258 | 1189 |
| PPV6-4R | AATAGCACCAAGGGGTCTCGTAG |  |  |
| PPV6-5F | TCTACGAGACCCCTTGGTGCTAT | 4235～5723 | 1489 |
| PPV6-5R | CCATCTTGATCGAGAATGAAAGC |  |  |
| PPV6-6F | AAAAGTCCCCTGCCTGTTTAGAGTCCCA | 5378～6056 | 679 |
| PPV6-6R | GTCAACTGCTATAACCGTGATATTGCGT |  |  |
