# Supplementary material for: A retrospective study on the prevalence and genetic characteristics of porcine parvovirus 6 in Guangxi, China
Source: Front Microbiol. 2026 Jan 27;17:1754811. doi: 10.3389/fmicb.2026.1754811 (PMC12886342; doi:10.3389/fmicb.2026.1754811)
Supplement: Supplementary file 4 [file Table_2.docx]

**Table S2**: Summary of reference sequences included in this study

| Strain name | GenBank Accession | Year | Country | Genera |
| --- | --- | --- | --- | --- |
| Bovine AAV | NC005889 | 2003 | USA | Dependoparavovirus |
| AAV-1 | NC002077 | 1998 | USA | Dependoparavovirus |
| DA-1 | NC006263 | 2004 | USA | Dependoparavovirus |
| Goose parvovirus | NC001701 | 1995 | USA | Dependoparavovirus |
| P15-1 | KX384823 | 2016 | Poland | Copiparvovirus |
| KSU1-AZ-2014 | KR709262 | 2015 | USA | Copiparvovirus |
| TJ | KF999685 | 2012 | China | Copiparvovirus |
| U18-9 | KX384817 | 2016 | Poland | Copiparvovirus |
| PPV-4 | NC014665 | 2009 | USA | Copiparvovirus |
| PPV-5 | JX896318 | 2012 | USA | Copiparvovirus |
| Bovine parvovirus 2 | NC006259 | 2001 | USA | Copiparvovirus |
| CnPPV_YW8 | GU938299 | 2009 | China | Tetraparvovirus |
| Human parvovirus 4 | NC007018 | 2004 | USA | Tetraparvovirus |
| Human parv4 G2 | DQ873391 | 2006 | USA | Tetraparvovirus |
| HK1 | EU200669 | 2007 | China | Tetraparvovirus |
| HK-S04 | JF504700 | 2011 | China | Tetraparvovirus |
| Porcine hokovirus | EU200671 | 2007 | China | Tetraparvovirus |
| PPV3-DJH14 | MK092411 | 2018 | China | Tetraparvovirus |
| ADV | NC001662 | 2000 | USA | Amdoparvovirus |
| PPV | NC001718 | 2000 | USA | Protoparvovirus |
| Minute virus of mice | NC001510 | 2000 | USA | Protoparvovirus |
| Canine parvovirus | NC001539 | 2000 | USA | Protoparvovirus |
| FVP-3.us_67 | EU659111 | 2008 | USA | Protoparvovirus |
| Human erythrovirus V9 | NC004295 | 2002 | USA | Erythroparvovirus |
| Simian parvovirus | U26342 | 1995 | USA | Erythroparvovirus |
| TuPV JO11 | KM598421 | 2014 | USA | Unclassified |
| ABU-P1 | GU214704 | 2009 | USA | Unclassified |
| ParvoD62/2013 | KM254172 | 2012 | Korea | Unclassified |
| Bovine parvovirus | NC001540 | 2000 | USA | Bocaparvovirus |
| Primate bocaparvovirus 1 | NC007455 | 2005 | USA | Bocaparvovirus |
| GD-2014-1 | KY996756 | 2014 | China | Chapparvovirus |
| 42 | KU563733 | 2015 | USA | Chapparvovirus |
| GD-2014-2 | KY996757 | 2014 | China | Chapparvovirus |
| GD-2014-3 | KY996758 | 2014 | China | Chapparvovirus |
| Galleria mellonella densovirus | NC004286 | 2003 | USA | Unclassified |
| KSU4-NE-2014 | KR709265 | 2014 | USA | Copiparvovirus |
| 3456/2012 | MH558679 | 2012 | Spain | Copiparvovirus |
| SK-CA-2018 | MH820262 | 2018 | Canada | Copiparvovirus |
| KSU7-SD-2014 | KR709268 | 2014 | USA | Copiparvovirus |
| K13-8 | KX384813 | 2016 | Poland | Copiparvovirus |
| K17-3 | KX384821 | 2016 | Poland | Copiparvovirus |
| K17-10 | KX384820 | 2016 | Poland | Copiparvovirus |
| K13-4 | KX384819 | 2016 | Poland | Copiparvovirus |
| PPV6-KF4 | MH447538 | 2017 | South Korea | Copiparvovirus |
| U18-1 | KX384822 | 2016 | Poland | Copiparvovirus |
| U18-7 | KX384815 | 2016 | Poland | Copiparvovirus |
| U18-8 | KX384816 | 2016 | Poland | Copiparvovirus |
| KSU1-AZ-2014 | KR709262 | 2014 | USA | Copiparvovirus |
| 17BKWB09 | MW711840 | 2017 | South Korea | Copiparvovirus |
| KSU3-KS-2014 | KR709264 | 2014 | USA | Copiparvovirus |
| Br | KY094494 | 2008 | Brazil | Copiparvovirus |
| KSU6-IA-2014 | KR709267 | 2014 | USA | Copiparvovirus |
| AH-PPV620178-1 | MW853954 | 2017 | China | Copiparvovirus |
| KSU2-AZ-2014 | KR709263 | 2014 | USA | Copiparvovirus |
| U18-9 | KX273435 | 2014 | Poland | Copiparvovirus |
| 171220-44 | MH921909 | 2017 | South Korea | Copiparvovirus |
| 171220-11 | MH921907 | 2017 | South Korea | Copiparvovirus |
| 17BKWB41 | MW711841 | 2017 | South Korea | Copiparvovirus |
| FJ2017 | MG760726 | 2017 | China | Copiparvovirus |
| JS | KF999683 | 2012 | China | Copiparvovirus |
| BJ2 | KF999682 | 2012 | China | Copiparvovirus |
| AH-PPV620178-2 | MW853955 | 2017 | China | Copiparvovirus |
| AH-PPV620178-3 | MW853956 | 2017 | China | Copiparvovirus |
| BJ1 | KF999681 | 2013 | China | Copiparvovirus |
| SC | KF999684 | 2012 | China | Copiparvovirus |
